# Safety of Pirfenidone in Patients with Idiopathic Pulmonary Fibrosis: Experience from 92 Sites in an Open-Label US Expanded Access Program

**DOI:** 10.1007/s41030-017-0049-z

**Published:** 2017-09-06

**Authors:** Lisa Lancaster, Lake Morrison, Alexander Auais, Beiying Ding, Ahmar Iqbal, Boris Polman, Kevin R. Flaherty

**Affiliations:** 1 Department of Medicine, Vanderbilt University, Nashville, TN USA; 2 Department of Medicine, Duke University, Durham, NC USA; 3 Genentech, Inc., South San Francisco, CA USA; 4 Department of Internal Medicine, University of Michigan, Ann Arbor, MI USA

**Keywords:** Idiopathic pulmonary fibrosis, Pirfenidone, Safety

## Abstract

**Introduction:**

In phase 3 clinical trials, pirfenidone significantly slowed disease progression with a well-defined and medically manageable safety profile in patients with idiopathic pulmonary fibrosis (IPF). This study examined safety events related to pirfenidone in patients with IPF in an expanded access program in the US.

**Methods:**

The Expanded Access Program allowed patients with IPF access to pirfenidone prior to US Food and Drug Administration approval. Patients had an IPF diagnosis including a definite or possible usual interstitial pneumonia (UIP) pattern, predicted forced vital capacity ≥50%, and predicted diffusing capacity for carbon monoxide ≥30%. Clinical laboratory data and adverse drug reactions (ADRs) deemed causally related to pirfenidone were analyzed using descriptive summary statistics.

**Results:**

Of the 1620 patients treated, 1221 (75.4%) completed the program: 66.5% had definite UIP, and 33.2% had possible UIP. Mean (SD) pirfenidone exposure was 22.8 (9.6) weeks, and mean (SD) daily dose during the course of treatment was 2058.7 (399.2) mg. ADRs occurred in 64.9% of patients: 3.3% were severe and 0.2% life threatening. The most common ADRs were nausea (22.6%) and fatigue (19.6%); 13.0% of patients discontinued due to ADRs. Serious ADRs occurred in 24 patients (1.5%), which were primarily related to elevated liver function enzymes (ten patients, 0.6%). No ADRs led to death.

**Conclusions:**

In this open-label study of 1620 patients with IPF, including those with possible UIP, the safety profile of pirfenidone was consistent with that of earlier clinical trials, and no new safety signals were identified. NCT02141087.

**Funding:**

Genentech, Inc., a member of the Roche group.

## Introduction

Idiopathic pulmonary fibrosis (IPF) is a progressive, unpredictable, and irreversible fibrosing lung disease with median survival rates estimated at 2–5 years from the time of diagnosis [1–4]. Pirfenidone is an oral antifibrotic agent whose clinical efficacy and safety have been demonstrated in three multinational, randomized controlled phase 3 trials [5, 6]. In these trials, gastrointestinal and skin-related events were the most common adverse events (AEs), which were generally mild to moderate in severity and rarely resulted in treatment discontinuation [5–7]. Furthermore, integrated safety analyses of the clinical trials demonstrated that long-term treatment (up to 9.9 years) with pirfenidone is generally well tolerated [8, 9]. However, data on the safety and tolerability of pirfenidone in patients with IPF in clinical practice in the US remain limited.

To further evaluate the safety of pirfenidone in patients with IPF in real-world clinical practice in the US, ADRs were assessed from this multicenter, open-label, single-arm Expanded Access Program (EAP) designed to provide pirfenidone to patients with IPF in the US prior to its approval by the US Food and Drug Administration (FDA).

## Methods

### Study Patients

Patients with a clinical and radiographic diagnosis of IPF, including the presence of usual interstitial pneumonia (UIP) pattern or possible UIP pattern based on high-resolution computed tomography (HRCT), were included. Patients had to have predicted forced vital capacity (FVC) ≥50% and predicted diffusing capacity for carbon monoxide (DL_CO_) ≥30%. Patients were excluded if they were receiving an investigational agent for IPF (prior pirfenidone use was permitted), and, because tobacco cigarette smoking increases levels of CYP1A2 (associated with increased pirfenidone clearance), patients were excluded if they had smoked cigarettes within 3 months prior to screening or were unwilling to avoid tobacco products during the study [10, 11]. All procedures followed were in accordance with the ethical standards of the responsible committee on human experimentation (institutional and national) and with the Helsinki Declaration of 1964, as revised in 2013. All investigators obtained institutional review board approval for the investigation and all patients provided informed consent. The Clinica Trials.gov registration number for this study is NCT02141087.

### Study Design

The study period was from April 14, 2014, to May 15, 2015. As specified in the protocol, the access program ended ≈6 months after pirfenidone became commercially available in the US, following approval by the US FDA.

All patients were initially prescribed pirfenidone 2403 mg/day in three equally divided doses. If a patient prematurely discontinued pirfenidone before it was commercially available, they were evaluated within 28 days after the last dose of treatment or the date on which the decision to discontinue was made. If a patient transitioned directly to the commercial drug, then the follow-up visit took place at the time of transition or within 1 week after the last dose of the study drug.

### Analyses

ADRs deemed to be related to pirfenidone by the investigator/site were collected and reported. Outcome measures were ADRs, serious ADRs (SADRs), severe or life-threatening ADRs, ADRs resulting in early discontinuation of pirfenidone, ADRs with outcome of death, and changes in clinical laboratory parameters, which were assessed at weeks 1, 2, 4, 8, 12, 16, 20, and 26 and every 13 weeks thereafter. SADRs were defined as serious AEs deemed to be causally related to pirfenidone. The severity of ADRs was recorded as mild, moderate, severe, or life threatening based on the National Cancer Institute’s Common Terminology Criteria for Adverse Events version 4.0 (NCI-CTCAE v4.0). Laboratory data were graded using the NCI-CTCAE v4.0 toxicity grade. Liver function tests were conducted at screening; at weeks 2, 4, 8, 12, 16, 20, and 26; every 13 weeks thereafter; and at follow-up after discontinuation. No formal hypothesis testing was conducted; descriptive summary statistics are provided. Descriptive statistics for continuous variables include the number of patients (*N*), mean and SD, median, minimum, and maximum. Unless otherwise indicated, descriptive statistics were calculated based on patients with nonmissing data.

## Results

### Baseline Characteristics and Patient Disposition

A total of 1620 patients with IPF were enrolled in the EAP, who were treated at 92 sites in the US. The majority of patients were white (95.8%) and male (74.7%). The mean (SD) age was 71.0 (7.7) years; one third of patients were ≥75 years of age, including 211 patients (13.0%) who were ≥80 years of age (Table 1). At the time of diagnosis, 1077 patients (66.5%) had definite UIP and 538 (33.3%) had possible UIP per HRCT assessment (no HRCT data were available for five patients). Baseline mean (SD) values for percent predicted FVC and percent predicted DL_CO_ were 70.3 (14.4) and 48.2 (14.1), respectively.

**Table 1:** Patient demographics and baseline characteristics

Characteristic	Pirfenidone (*N* = 1620)
Age, mean (SD), years	71.0 (7.7)
<65	294 (18.1)
≥65 to <75	787 (48.6)
≥75 to <80	328 (20.2)
≥80	211 (13.0)
Male, *n* (%)	1210 (74.7)
White, *n* (%)	1552 (95.8)
BMI, mean (SD), m^2^	29.5 (5.1)
Weight, mean (SD), kg	87.2 (17.5)
HRCT assessment of UIP, *n* (%)	
Definite	1077 (66.5)
Possible	538 (33.2)
FVC, mean (SD), % predicted	70.3 (14.4)
DL_CO_, mean (SD), % predicted	48.2 (14.1)

*DL*_*CO*_ diffusing capacity for carbon monoxide, *FVC* forced vital capacity, *HRCT* high-resolution computed tomography, *UIP* usual interstitial pneumonia

Of the 1620 patients, 1221 (75.4%) completed the program and 399 (24.6%) discontinued early; 210 patients (13.0%) discontinued due to ADRs/SADRs. Other reasons for discontinuation are described in S1 Figure.

### Pirfenidone Exposure

Overall, the mean (SD) duration of prescribed pirfenidone treatment was 22.8 (9.6) weeks. The majority of patients (77.7%) received pirfenidone for 12–36 weeks, 7.6% for 36 to <48 weeks, 8.3% for 6 to <12 weeks, 5.5% for 2 to <6 weeks, and 0.9% for >0 to <2 weeks. The mean (SD) daily prescribed dose was 2058.7 (399.2) mg.

In total, 364 patients (22.5%) had ≥1 prescribed dose reduction; one patient had >5 dose reductions. There were 275 reported dose interruptions (defined as a gap of ≥1 day between nonzero doses with a prescribed dose of zero) in 227 patients (14.0%); 187 patients (11.5%) had one interruption, 32 (2.0%) had two interruptions, eight (0.5%) had three interruptions, and no patient had >3 prescribed dose interruptions. The mean (SD) duration of dose interruptions was 13.4 (12.2) days; dose interruptions of ≥14 days were reported in 97 patients (6.0%). For patients who had dose reductions, the mean (SD) time to the first dose reduction was 1.9 (1.5) months. For patients who had dose interruptions, the mean (SD) time to the first dose interruption was 2.1 (1.6) months.

### Adverse Drug Reactions

Of the 1620 treated patients, 1051 (64.9%) experienced ≥1 ADR (Table 2). The most common ADRs were nausea (22.6%), fatigue (19.6%), and diarrhea (11.2%). Most ADRs were mild to moderate in severity. Dose modification or interruption due to an ADR occurred in 377 patients (23.3%). No ADRs resulted in death. SADRs (defined as serious AEs deemed causally related to pirfenidone) were reported in 24 patients (1.5%); an SADR led to dose modification or interruption in five patients (0.3%) and to treatment discontinuation in 17 patients (1.0%). The most common SADRs were elevated alanine aminotransferase (ALT) and elevated hepatic enzyme, which were reported in five patients (0.3%) each. Of the ADRs that led to early discontinuation of pirfenidone, most were mild to moderate in severity. The most common ADRs resulting in treatment discontinuation were nausea (2.7%), fatigue (1.9%), and rash (1.4%) (S2 Table). Diarrhea, reported in 182 patients (11.2%), resulted in treatment discontinuation in five patients (0.3%). Photosensitivity reactions, reported in 52 patients (3.2%), resulted in treatment discontinuation in five patients (0.3%).

**Table 2:** Overview of ADRs

	Pirfenidone (*N* = 1620)
ADRs, *n* (%)	
Patients with ≥1 ADR	1051 (64.9)
ADRs with >5% incidence	
Nausea	366 (22.6)
Fatigue	317 (19.6)
Diarrhea	182 (11.2)
Rash	133 (8.2)
Anorexia	116 (7.2)
Dyspepsia	112 (6.9)
Dizziness	99 (6.1)
Decreased appetite	95 (5.9)
Gastroesophageal reflux disease	88 (5.4)
ADR resulting in death	0
ADR leading to dose modification or interruption	377 (23.3)
ADR leading to discontinuation of pirfenidone	210 (13.0)
SADRs, *n* (%)^a^	
Patients with ≥1 SADR	24 (1.5)
SADRs in ≥2 patients	
ALT elevated^b^	5 (0.3)
Hepatic enzyme elevated^c^	5 (0.3)
AST elevated^b^	2 (0.1)
Diarrhea	2 (0.1)
Nausea	2 (0.1)
SADR resulting in death	0
SADR leading to dose modification or interruption	5 (0.3)
SADR leading to discontinuation of pirfenidone	17 (1.0)

*ADR* adverse drug reaction, *ALT* alanine aminotransferase, *AST* aspartate aminotransferase, *LFT* liver function test, *SADR* serious adverse drug reaction, *ULN* upper limit of normal

^a^SADR is defined as a serious adverse event deemed to be causally related to pirfenidone

^b^Two patients had both elevated ALT and AST levels

^c^Preferred Term used for events not coded to other Preferred Terms; these included the following investigator-specified verbatim terms: elevated AST and ALT, elevated AST/ALT, elevated LFTs >5 ULN, elevated liver enzymes, high ALT/AST, increased ALT and AST, increased ALT and AST labs, and increased liver enzymes

**Fig. 1:**
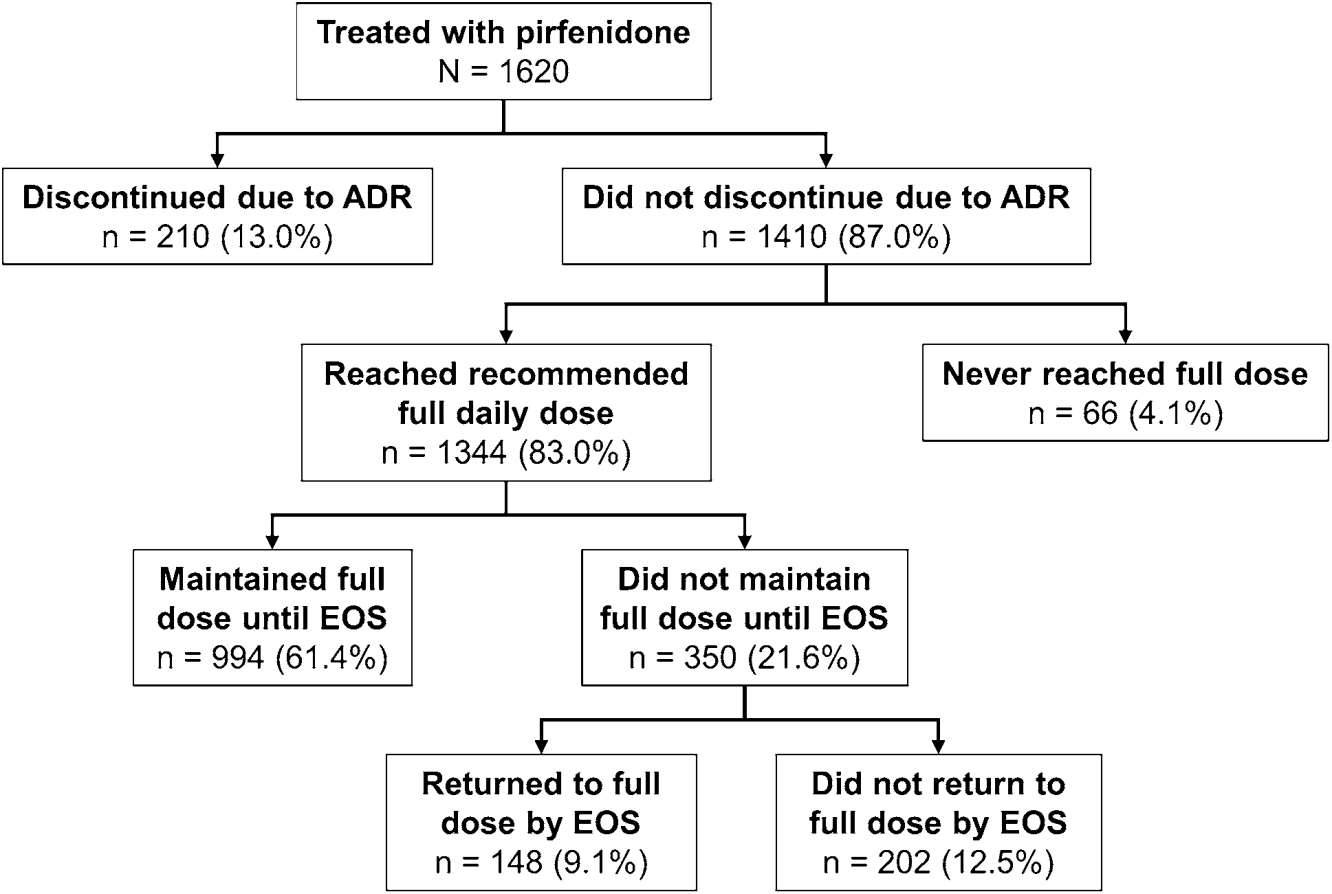
Pirfenidone dosing and dose reductions/interruptions. Percentages are of the total population (*N* = 1620). *ADR* adverse drug reaction, *EOS* end of study

Of the 1410 patients who did not discontinue due to an ADR, 1344 (95.3%) reached the recommended full daily dose of 2403 mg (Fig. 1); the mean (SD) time to full dose in these patients was 16.6 (9.8) days. For patients who did not discontinue due to an ADR, 994 (70.5%) reached and maintained the full dose until the end of the study and 350 (24.8%) reached the full dose but also experienced a dose reduction or interruption; nearly half of these patients [148 (42.3%)] returned to the full dose by the end of the study.

**Fig. 2:**
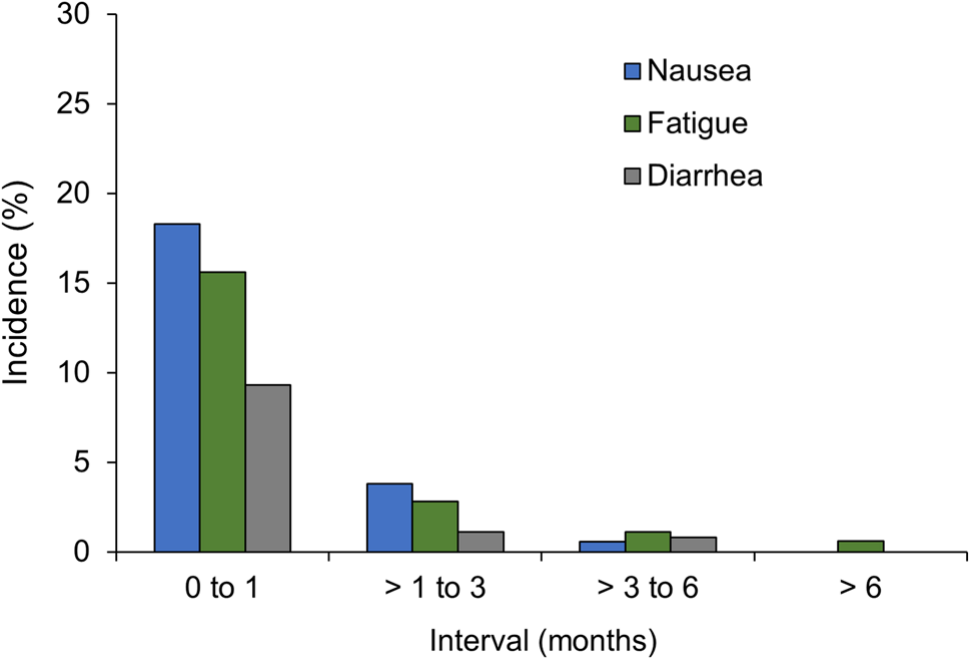
Incidence of adverse drug reactions: new-onset nausea, fatigue, or diarrhea

Most ADRs tended to first occur early in the course of treatment (Fig. 2). Of the 366 patients with an ADR of nausea, 296 experienced it within the first month; the mean (SD) time to new-onset nausea was 0.70 (0.88) months. Similarly, all 182 patients with an ADR of diarrhea experienced it by month 6 (151 patients experienced it within the first month); the mean (SD) time to new-onset diarrhea was 0.74 (1.05) months. Of the 317 patients who experienced fatigue as an ADR, 316 did so by month 6 and 253 did so within the first month; the mean (SD) time to new-onset fatigue was 0.83 (1.10) months.

Severe ADRs (defined as those causing marked limitation in activity, some assistance usually required, medical intervention/therapy required, hospitalization possible; NCI-CTCAE v4.0) were reported in 53 patients (3.3%); the most common severe ADRs were fatigue and nausea in nine patients (0.6%) each (S3 Table). Of the patients who had severe nausea, four discontinued treatment, one had a permanent dose reduction, one had a temporary dose interruption, and three had no action taken (with respect to the drug). Of the patients who had severe fatigue, one discontinued treatment, four had a permanent dose reduction, three had a temporary dose reduction, and one had no action taken (with respect to the drug). Life-threatening ADRs were reported in three patients (0.2%): coagulopathy, IPF, and lung squamous cell carcinoma (stage unspecified) in one patient each.

There was a slight age-related increase in the proportion of patients with ≥1 ADR (Table 3). The proportion of patients with ADRs resulting in dose modification/interruption or discontinuation also increased with increasing age: an ADR leading to dose modification/interruption occurred in 32.7% of patients aged ≥80 years and in 18.0% of patients aged <65 years; an ADR leading to discontinuation occurred in 20.9% of patients aged ≥80 years and in 7.5% of patients aged <65 years. The proportions of patients with ≥1 SADR or with a dose modification/interruption or discontinuation due to an SADR were comparable across age groups.

**Table 3:** ADRs stratified by age

*n* (%)	<65 years (*n* = 294)	≥65 to <75 years (*n* = 787)	≥75 to <80 years (*n* = 328)	≥80 years (*n* = 211)
Patients with ≥1 ADR	181 (61.6)	501 (63.7)	217 (66.2)	152 (72.0)
ADR leading to dose modification or interruption	53 (18.0)	163 (20.7)	92 (28.0)	69 (32.7)
ADR leading to discontinuation of pirfenidone	22 (7.5)	86 (10.9)	59 (18.0)	43 (20.9)
Patients with ≥1 SADR	4 (1.4)	12 (1.5)	4 (1.2)	4 (1.9)
SADR leading to dose modification or interruption	1 (0.3)	2 (0.3)	1 (0.3)	1 (0.5)
SADR leading to discontinuation of pirfenidone	3 (1.0)	8 (1.0)	4 (1.2)	2 (0.9)

*ADR* adverse drug reaction, *SADR* serious ADR

Overall, the safety profile was similar between patients with definite or possible UIP (Table 4), with ≥1 ADR reported in 63.8% of patients with definite UIP and in 67.1% of patients with possible UIP. The proportion of patients with ≥1 ADR leading to discontinuation was also comparable between the groups, occurring in 12.0% of patients with definite UIP and in 14.9% of patients with possible UIP.

**Table 4:** ADRs stratified by patients with definite or possible UIP by HRCT

*n* (%)	Definite UIP (*N* = 1077)	Possible UIP (*N* = 538)
Patients with ≥1 ADR	687 (63.8)	361 (67.1)
ADR leading to dose modification or interruption	261 (24.2)	115 (21.4)
ADR leading to discontinuation of pirfenidone	129 (12.0)	80 (14.9)
Patient with ≥1 SADR	14 (1.3)	10 (1.9)
ADR with >5% incidence^a^		
Nausea	229 (21.3)	137 (25.5)
Fatigue	219 (20.3)	97 (18.0)
Diarrhea	119 (11.0)	63 (11.7)
Rash	88 (8.2)	45 (8.4)
Anorexia	82 (7.6)	34 (6.3)
Dyspepsia	74 (6.9)	38 (7.1)
Dizziness	60 (5.6)	39 (7.2)
Decreased appetite	61 (5.7)	33 (6.1)
Gastroesophageal reflux disease	54 (5.0)	34 (6.3)

*ADR* adverse drug reaction, *HRCT* high-resolution computed tomography, *SADR* serious ADR, *UIP* usual interstitial pneumonia

^a^Sorted in descending order of frequency in the overall population

### Laboratory Parameters

Overall mean increases in ALT levels were small and transient. Thirteen patients (0.8%) had postbaseline grade 3 elevations in ALT (two had grade 1 elevation at baseline), and no patients had grade 4 elevations. Four patients (0.2%) had grade 3 elevations in aspartate aminotransferase (AST) levels, three of whom had grade 1 elevations at baseline; no patients had grade 4 elevations in AST. The majority of grade 3 elevations for ALT and AST occurred at week 8. All postbaseline alkaline phosphatase, bilirubin, and creatinine levels were grade 2 or lower.

## Discussion

This study evaluated safety outcomes in a large cohort of patients with IPF treated with pirfenidone in an outpatient clinical setting at daily doses up to 2403 mg (801 mg 3 times/day) for up to 47.1 weeks [mean (SD) 22.8 (9.6) weeks]. Most patients (75.4%) completed the program; 61.4% of patients reached and maintained the full daily dose until the end of the study. ADRs were mostly mild to moderate in severity and led to treatment discontinuation in 13% of patients. No ADRs resulted in death, and no new safety signals were identified. Overall, the safety profile of pirfenidone in patients with IPF in an open-label, real-world setting at daily doses up to 2403 mg was similar to that reported in controlled clinical trials.

Consistent with findings from clinical trials, the most common ADRs in this study were nausea (22.6%), fatigue (19.6%), and diarrhea (11.2%). A recent integrated safety analysis of four pirfenidone clinical trials reported nausea, fatigue, and diarrhea as treatment-emergent AEs in 37.6, 28.2, and 28.1% of patients, respectively [8]. In addition, the incidences of the most common ADRs resulting in discontinuation—nausea (2.7%), fatigue (1.9%), and rash (1.4%)—were consistent with those in the clinical trials: in the integrated safety analysis of pirfenidone trials, nausea and rash led to treatment discontinuation in 1.7% and 2.2% of patients, respectively [8]. In this EAP, the majority of the common ADRs (nausea, fatigue, and diarrhea) started within the first month of treatment. Consistently, a recent safety assessment of patients with IPF treated with pirfenidone in the clinical trials showed that onset of these ADRs decreased markedly after the first 6 months and remained low thereafter (up to 54 months) [9].

Among patients who reached the full 2403 mg/day pirfenidone dose and had a dose reduction or interruption (and did not discontinue due to an ADR), slightly less than half were able to return to the full dose by the end of the study, suggesting that dose modification may have been an effective strategy for continuing treatment. Dose modification or interruption due to an ADR was reported in 23% of patients, which likely allowed patients to continue treatment and contributed to the low discontinuation rate. Consistent with this, a recent publication of an expert panel discussion recommended dose reduction and temporary dose interruption as effective methods to manage GI and skin-related AEs in patients receiving pirfenidone [12].

The outpatient clinical population of patients with IPF in this study is reflected by the inclusion of approximately one third of patients with possible UIP per HRCT assessment and one third of patients aged ≥75 years (and 13% of patients aged ≥80 years). Real-world data on pirfenidone are limited; the data presented here are reassuring and consistent with data from the ongoing PASSPORT registry study [13]. Similar to the findings here, the most common ADRs reported in PASSPORT were nausea (15.7%) and fatigue (15.3%) over a mean of 6.3 months of exposure. Furthermore, 16% of patients in PASSPORT discontinued treatment due to an ADR, similar to the 13% of patients who discontinued treatment due to an ADR in this study. Although older patients (≥75 years) had a slightly higher but comparable incidence rate of ADRs, they tended to have higher rates of ADRs leading to discontinuation or dose reduction/interruption, particularly those aged ≥80 years. Although this may have been due to a higher severity of the ADR in older patients, other possible reasons include a higher number of comorbidities and of concomitant medications in older patients and more cautious treatment of older patients by physicians.

The strengths of this study come from the well-defined large cohort of patients. Interestingly, while this EAP reflects a real-life cohort of patients with limited exclusion criteria (including one third of patients with possible UIP), the observations are similar to those from controlled and more stringently managed clinical trials in which most patients had definite UIP. Although most ADRs were observed within the first 6 months, this study was limited by its relatively short duration, and studies with longer follow-up times are needed to confirm the long-term safety, tolerability, and use of pirfenidone treatment in real-world clinical practice.

## Conclusions

In this open-label, outpatient setting of patients with IPF, including 33.2% of patients with possible UIP and 13% of patients aged ≥80 years, the safety profile of pirfenidone was consistent with that of the controlled clinical trials, and no new safety signals were identified. Patients aged ≥80 years tolerated pirfenidone; however, dose modification may be more common in this group. Dose modification (including slow titration), lower doses, and dose interruptions may be effective ways to maintain treatment in patients who experience ADRs.
